# Low‐Level Arsenic Removal from Drinking Water

**DOI:** 10.1002/gch2.201700047

**Published:** 2018-11-04

**Authors:** Fatemeh Makavipour, Richard M. Pashley, A. F. M. Mokhlesur Rahman

**Affiliations:** ^1^ School of Physical Environmental and Mathematical Sciences UNSW Canberra Northcott Drive Canberra ACT 2610 Australia

**Keywords:** arsenic, cysteine, cystine, flotation, surfactants

## Abstract

The reported ability of cysteine and cystine to bind typical arsenic oxy‐ions in water is used as a basis for a study of the potential for using a surfactant with a cysteine head‐group for selective arsenic binding and removal in an ion flotation process. Several different head‐group attachment methods are studied with cysteine and cystine and with single‐ and double‐chain surfactants. A comparison of the properties of these surfactants with some other surface‐active compounds, with groups like those on cysteine, suggest that few compounds have suitable characteristics for the efficient removal of low levels of arsenic from drinking water. An amino‐acid‐based single‐chain surfactant is synthesized by reacting cysteine with octanoyl chloride to obtain octanoyl cysteine, which is then used in a study of selective ion flotation for the removal of low levels of arsenic from drinking water. This compound has high water solubility and causes extensive foaming in a typical flotation chamber and removed 99.4–99.9% of the 5 mg L^−1^ arsenic present in the contaminated water in a simple, single‐stage ion flotation process, using either air or nitrogen gas. These laboratory results indicate that these surfactants can be useful in the large‐scale treatment of low‐level arsenic‐contaminated water.

## 1 Introduction

Arsenic is a trace element which can be found in the earth's crust with an abundance of 54 and average concentration of about 5 mg L^−1^. Some processes such as mining, well drilling, and weathering^1^ could increase the released amount of this heavy metal to the environment, especially into ground waters, putting people's health at risk of cancer (mostly bladder, lung. and skin) and related diseases.[[qv: 1b,2]] Ground water contamination can also lead to agriculture products contamination, such as in rice.^3^ Inorganic forms of arsenic which are toxic include As(V) and As(III). As(V) mainly exist predominantly under oxidizing conditions as arsenate (e.g., first ionized and second ionized products of arsenic acid H_2_AsO_4_^−^ and HAsO_4_^2−^) while As(III) naturally exists as arsenous acid (H_3_AsO_3_) predominantly under reducing conditions in a pH range of 2–9.^4^ The World Health Organization (WHO) reported an arsenic maximum contamination level of 0.01 mg L^−1^ in drinking water.^5^ In consequence, using low‐cost natural adsorbents has become a matter of interest in research aimed at removing arsenic efficiently from water.^6^ In this study, *L‐*cysteine has been used as the adsorbent due to its natural amino acid structure and its existence in high‐level protein food and also the known interaction of its sulfhydryl groups with arsenic, through which it binds arsenic to specific proteins.[[qv: 1b]] In addition, *L‐*cysteine has been used as prereductant of As(V) to As(III) successfully in the literature^7^ and also there has been successful reports for arsenic removal from water using cysteine coated silica microparticles^8^ and also on cysteine itself.^9^

It has been reported[[qv: 1b]] that several cysteine molecules are required to bind to each arsenic ion species and hence it seems reasonable to assume that capture of arsenic onto cysteine groups bound to a solid substrate, which has been observed,^8^ might be enhanced by using a more fluid source of the cysteine groups. This could be achieved using surfactants with cysteine head‐groups adsorbed at the water–air interface that is, adsorbed onto a bubble surface. Rising bubbles within a flotation column could then offer a continuous supply of cysteine coated monolayers, where the surfactants and head‐groups will be relatively mobile, at room temperature, especially for surfactants with hydrocarbon chain lengths of 12 or less. Collisions between the dissolved ionic arsenic species and the cysteine coated rising bubbles might be useful for selective and efficient arsenic ion capture and removal in a one step, continuous water treatment process.

Surfactants generally adsorb rapidly and form monolayers on the surface of bubbles in water; they also have the remarkable ability to self‐assemble in aqueous solution at concentrations above the critical micelle concentration (CMC). The structures spontaneously formed by surfactants in solution are created to reduce the exposure of the hydrocarbon chains to water.^10^

In a typical conductivity graph versus concentration, there is a sharp transition that occurs in most of the solution properties which corresponds to the formation of self‐assembled structures called micelles. The concentration at which they are formed is a characteristic of the particular surfactant and is called the “critical micelle concentration,” or CMC.

Ionic surfactants form micelles when their hydrocarbon chains are sufficiently fluid, that is at temperatures above their Krafft temperature or their chain melting temperature. Below the Krafft temperature, the surfactant becomes insoluble rather than self assembles. Micelles are one type of aggregates and the formation of other types of aggregations or self‐assemblies depends on some parameters, such as physical constraints that normally arise from hydrocarbon chain volume and head‐group area. The flexibility of the chains and the intermolecular forces are also other effective parameters.

The formation of aggregates is a very important property of surfactants, which is of fundamental importance in their detergent cleaning action. The hydrocarbon regions in the aggregates solubilize fatty organic materials (dirt) during cleaning.

The degree of ionization of micelle, α, is a fraction of an ionic surfactant's counter ions which dissociate from the micelles, leaving the micelles charged. This value is crucial to understand many aspects of the behavior of micelles. Namely, reaction rates between organic substrates and hydrophilic ions that can “bind” to the micelle. Obviously, the value of α is an important element in micelle stability in general and in the growth of spherical micelles into rod‐like structures which can lead to viscoelastic behavior.^11^ Also, a complete thermodynamic or structural theory of micelles must be able to predict values of and changes in α, thus, accurate experimental values are needed. It is also likely that the degree of ionization will be the same for surfactant monolayer coated bubbles, used in the flotation process discussed in this work.

In this study, several double‐chain (denoted *D‐*) and single‐chain (denoted *S‐*) cysteine‐based surfactants (of carbon length chain of 8 and 12) were synthesized and their properties for use in a bubble/flotation system were investigated. As, amino acid‐based surfactants (cysteine‐surfactants) are considered more environmentally friendly and biodegradable^12^ each of the component groups in cysteine was also studied for their potential application in arsenic ion removal. These were amine, carboxylic acid, and thiol groups, which, in each case, were studied as potential surfactants in a flotation separation system. In addition, the three components were also tested as a physically combined mixture.

## 2 Results and Discussion

### 2.1 Surfactants Characterization

^1^H NMR spectra were obtained for samples of double recrystallized octanoyl‐cysteine. Typical spectrum is given in Figure S1 in the Supporting Information.

The spectra showed a sharp peak of mono deuerated water (HDO) at 4.70 ppm 1H NMR: δ (ppm) = 4.33 (t, 1H, CHCOONa), 2.80 (t, 1H, SCH_2_), 2.18 (m, 2H, COCH_2_), 1.47 (m, 2H, COCH_2_CH_2_), 1.30 (d, 8H, (CH_2_)_4_)), 0.71 (t, 3H, CH_3_). This confirms the structure of the C_11_H_21_NO_3_S in D_2_O and the expected proton interactions.

Fourier transform infra red (FT‐IR) spectrum was obtained for samples of double recrystallized octanoyl‐cysteine (as shown in Figure S2, Supporting Information), which gave (*ʋ*_max_, cm^−1^): 3421.72 (NH), 2920.23, 2854.65 (CH), 1624.06 (CO), 1504.48 (*ʋ*_ass_ CO^2−^), 1419.61 (*ʋ*_s_ CO^2−^). This spectral analysis confirms the presence of NH, COO, CO, and CH groups in the samples.

The elemental analysis for samples of recrystallized cysteine surfactants are shown in **Table** **1**. Octanoyl‐cysteine, C_11_H_21_NO_3_S, clearly, after two recrystallizations gave an almost exact match with the expected elemental analysis. A melting point of 127 °C was observed for samples of twice recrystallized octanoyl‐cysteine, which is close to the expected literature value of 131–133 °C.^13^

**Table 1:** Total elemental analysis (T.E.A.) of the synthesized surfactants after two times recrystallization (three times for *D‐*octanoyl‐cystine and *D‐*dodecanoyl‐cystine). *D* denotes double‐chain and *S* denotes single‐chain. MP: melting point

	Name	Formula	MP [°C]	T.E.A.	%C	%H	%N	%S
1	*S*‐octanoyl‐cysteine	C_11_H_21_NO_3_S	127.0	Theory	53.41	8.56	5.66	12.96
				Found	53.58	9.04	5.56	12.91
2	*D*‐octanoyl‐cystine	C_22_H_40_N_2_O_6_S_2_	192.47	Theory	53.26	8.20	5.69	13.01
				Found	46.68	7.59	7.26	15.98
3	*S‐*dodecanoyl‐cysteine	C_15_H_29_NO_3_S	143.0	Theory	59.36	9.65	4.62	10.56
				Found	60.84	10.76	2.82	5.28
4	*D*‐dodecanoyl‐cystine	C_30_H_56_N_2_O_6_S_2_	215.05	Theory	59.55	9.35	4.63	10.60
				Found	47.36	9.16	3.66	7.50
5	*S*‐octyl isocyanate‐cysteine	C_12_H_24_N_2_O_3_S	90.80	Theory	52.13	8.79	10.14	11.60
				Found	71.84	13.96	10.01	0.76
6	*D*‐octyl isocyanate‐cystine	C_24_H_46_N_4_O_6_S_2_	90.70	Theory	52.32	8.43	10.17	11.64
				Found	71.84	14.16	10.07	0.61
7	*S*‐octyl‐cysteine	C_11_H_23_NO_2_S	206.30	Theory	56.60	9.95	6.00	13.74
				Found	59.10	10.54	5.55	12.50

For other surfactants, as can be seen in Table 1, after two to three recrystallizations compounds gave roughly the expected elemental analysis. However, for octyl isocyanate surfactants, it seems that the only element match was for nitrogen content and the other elements did not show anything close to expected values. It appears that the surfactant synthesis was not successful in achieving the desired octyl isocyanate surfactants, at least not for the double chain surfactants, which would also explain their complete insolubility. Also, there is a possibility that nonionic urea surfactants were produced, which are known to be highly insoluble in aqueous solutions.

### 2.2 Evaluation of Suitable Surfactants for Arsenic Removal

An investigation of the basic properties of several synthesized surfactants was carried out to determine suitable surfactants for the flotation system for removing arsenic; a summary of this study is shown in **Table** **2**.

**Table 2:** Physical properties and arsenic removal ability of several of the potential cysteine/cystine surfactants studied (note NT means not tested)

	Compound	Molecular formula	Solubility in water up to minimum 0.01 m	Foaming test	CMC [mol L^−1^]	Arsenic removal
1	*S*‐octanoyl cysteine	C_11_H_21_NO_3_S	Yes	Passed	0.11	99.4% to 99.9%(air)
2	*D*‐octanoyl cystine	C_22_H_40_N_2_O_6_S_2_	Yes	Passed	0.017	3%
3	*S*‐octyl cysteine	C_11_H_23_NO_2_S	Yes	Failed	0.006[[qv: 12a]]	0%
4	*S*‐octyl isocyanate cysteine	C_12_H_24_N_2_O_3_S	No	Failed	0.034^14^	NT
5	*D*‐octyl isocyanate cystine	C_24_H_46_N_4_O_6_S_2_	No	Failed	0.008^14^	NT
6	*S*‐dodecanoyl cysteine	C_15_H_29_NO_3_S	No (0.3 × 10^−3^ m)	Passed	0.05 × 10^−3^	NT
7	*D*‐dodecanoyl‐cystine	C_30_H_56_N_2_O_6_S_2_	No (0.2 × 10^−3^ m)	Passed	0.01 × 10^−3^	0%

Conductivity measurements of surfactant solutions over a range of concentrations were obtained for both single‐chain octanoyl cysteine and double‐chain octanoyl cystine in alkaline water (pH > 9) at 25 °C to determine their CMC values. For a simple monovalent ionizable surfactant, the conductivity slope with surfactant concentration should change (reduce) at the CMC. Some typical results are shown in Figures S3 and S4 in the Supporting Information, which clearly indicate fairly sharp transitions in slope. Single‐chain dodecanoyl cysteine and double‐chain dodecanoyl cystine had lower CMC values and lower solubility in water. Their CMC values, measured in a typical conductivity test in alkaline water, are shown in Figures S5 and S6 in the Supporting Information. The conductivity measurements were carried out above the Krafft Temperature of the double‐chain surfactant, which has been reported as higher than 30 °C.^15^ Even though both these surfactants had good foaming characteristics in a flotation cell, they were not soluble enough to be suitable surfactants for the removal of arsenic from water, especially in highly concentrated arsenic solutions. However, it was decided to test them for arsenic removal in the flotation process.

The degree of ionization of single‐chain octanoyl cysteine surfactant micelles can be estimated by measuring the change in slope of solution electrical conductivity (κ) versus total concentration (*C*_s_) as the solution goes through the CMC. At a solution pH of 8–9, the single‐chain octanoyl cysteine structure should be as shown in Figure 2.

Due to the large size of surfactant anion we can assume that its ion mobility is low; also given that *C*_s_ >> *C*_OH_^−^, as the C_OH_^−^ <10^−5^, therefore the solution electrical conductivity must have a direct relation with the concentration of sodium ions, as the contribution to conductivity of the surfactant anions and even the larger, charged micelles (with a large viscous drag) must be negligible. That means that the overall solution conductivity (κ) is only related to the concentration of free Na^+^ ions present in solution. Thus, we assume that

(1) $$\kappa \;\alpha \;\;C_{{\mathrm{Na}}^{+}}$$

Therefore, at surfactant concentrations (*C*_s_) below the CMC (*C*_s_ < CMC)

(2) $$\kappa =AC_{{\mathrm{Na}}^{+}}$$

and

(3) $$\kappa =AC_{s}$$

Assuming that, the *S‐*octanoyl‐cys monomer surfactant is always fully ionized, and that *A* is some constant. By comparison, at concentrations above the CMC (*C*_s_ > CMC) it must also be true that

(4) $$C_{{\mathrm{Na}}^{+}}=\mathrm{CMC}+\alpha \left(C_{s}-\mathrm{CMC}\right)$$

where α is the degree of ionization of the micelle, which is assumed to be independent of concentration. Consequently, above the CMC

(5) $$\kappa =A\left[\mathrm{CMC}+\alpha \left(C_{s}-\mathrm{CMC}\right)\right]$$

The gradients above and below the CMC could be calculated considering Equations (2) and (4), therefore at *C*_s_ > CMC

(6) $$\frac{d\kappa }{dC_{s}}=\alpha A$$

and at *C*_s_ < CMC

(7) $$\frac{d\kappa }{dC_{s}}=A$$

So, obviously, the ratio of these slopes gives the degree of ionization of the micelles above the CMC as follows, which for *S*‐octanoyl‐cys surfactant, is 0.47, from the data given in Figure S3 in the Supporting Information. Thus, we expect about 47% of the sodium ions dissociated from the micelles and the similar % from the surface of air bubbles coated with adsorbed surfactant molecules, at concentrations above the CMC.

The single‐chain octyl based cysteine surfactant, made from refluxing octyl bromide, failed the foaming test but was still used in the flotation cell due to its good solubility and high CMC. However, no arsenic removal was observed. Single‐chain octyl isocyanate cysteine and double‐chain octyl isocyanate cystine had acceptable CMC^14^ values but produced no foam in solution and were not very soluble in water. This was not expected given the comments in refs. 14 and,^16^ in which the synthesis of these surfactants was done similarly; a solubility of up to 0.2 m in water was quoted.[[qv: 16b,c]] However, several unsuccessful attempts were made to synthesize this surfactant and in each case a polymeric product was obtained, with no foaming and low water solubility.

For comparison, mercaptan and C_14_–TAB mixtures and mixtures of the three functional groups present in cysteine, but attached to individual short (C_8_–C_10_) hydrocarbon chains, also passed the foaming test and so were also used in flotation experiments for arsenic removal.

### 2.3 Arsenic Removal Process Using Surfactants

As can be seen in **Table** **3**, Tests 1 and 2, which were carried out using purified (twice recrystallized) single‐chain octanoyl cysteine, showed an average removal of 99% after 30 min. It is worth noting that by purification, not only did the efficiency of removing arsenic increase but also the removal occurred over a shorter time, 30 min rather than 90 min.

**Table 3:** Results of As analysis using inductively coupled plasma mass spectrometry (ICP‐MS) of the flotation process for different amino‐acid‐based surfactants and mixtures of cysteine functional groups in 100 mL of arsenic solution. The relative standard deviations for the measurements are given in brackets. In all Tests nitrogen was used instead of Test 2

No.	Compounda) [m]	As [mg L^−1^] after 30 min	As [mg L^−1^] after 60 min	As [mg L^−1^] after 90 min	Removal % after 30 min
1	*S*‐octanoyl cysteine (0.01)	0.029 (1.10)	0.032 (10.12)	0.031 (4.04)	99.4%
2	*S*‐octanoyl cysteineb) (0.01)	0.137 (1.03)	0.006 (0.61)	0.006 (0.60)	99.9%
3	*D*‐octanoyl cystine (0.001)	4.85 (0.33)	5.15 (1.70)	5.30 (8.43)	3.00%
4	*S*‐octyl cysteine (0.001)	5.10 (1.25)	5.24 (1.10)	5.31 (0.84)	0.0%
		0.54c) (1.07)	–	–	0.0%
5	*D*‐dodecanoyl cystine (0.01 × 10^−3^)	5.55 (2.04)	5.74 (1.60)	6.12 (1.85)	0.0%
6	Mercaptan and C_14_–TAB (0.001)	4.28 (3.84)	4.36 (1.46)	4.29 (0.72)	14.4%
7	Mixture of three functional groups (0.001)	1.03 (3.87)	–	–	0.0%

^a)^ Tests 1–6 with 5 mg L^−1^ arsenic solutions; Test 7 with 1 mg L^−1^ arsenic solution

^b)^ Test 2 air was used instead of nitrogen gas

^c)^ Test 4, second line: 0.5 mg L^−1^ arsenic solution.

At the beginning of the process, the level of bubbling solution plus foam was about 30 cm. After half an hour, this level went down to about 15 cm, so that the soap bubbles bursting on top of the column solution released any adsorbed arsenic back into the solution, hence preventing any further arsenic removal. In the all the ion flotation tests in this work, the maximum solution loss, carried over in the foam, was about 10–20% of the initial solution.

The mixture of sodium octanoate, *tert*‐dodecyl mercaptan, and octylamine, used as a model for the three functional groups on cysteine, did not show any arsenic adsorption after 30 min, even though there was significant foaming and foam carryover (20%). By comparison, the mixture of *tert*‐dodecyl mercaptan and C_14_–TAB showed modest (14%) adsorption of arsenic from 5 mg L^−1^ solution (after 30 min) with significant foaming and carryover (10%). This suggests that the thiol group has a significant role in arsenic adsorption in cysteine, as arsenic cannot be adsorbed onto the quaternary ammonium groups.^8^ The Quat surfactant was added to produce foaming, as the mercaptan alone did not foam. These results suggest that molecular structure of cysteine has a specific activity for arsenic adsorption.

**Figure 1:**
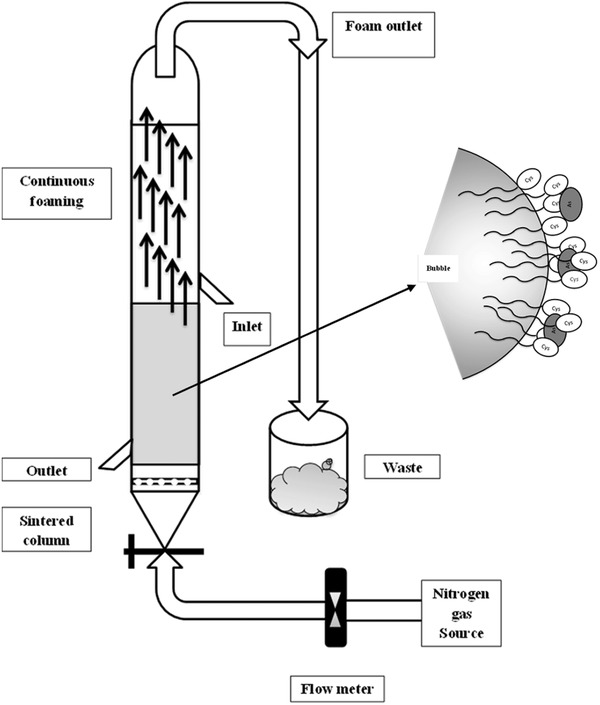
Schematic diagram of the column setup for the flotation process; dried air was also used as the gas for *S‐*octanoyl‐cys.

The process of surfactants attaching to a bubble surface while binding to arsenic species is shown in the schematic diagram in **Figure** **1**.

It seems that, after about half an hour, most of the arsenic separation had occurred and little or no further foam collection was obtained. For the *S*‐octanoyl‐cys experiments, there was little difference between dry air and dry N_2_, although the latter did help to stabilize the pH at around 8, because of the lack of carbon dioxide. It is assumed here that the natural oxidation of octanoyl cysteine will be similar to cysteine, that is, concentrated stock solutions can be stored because of the low levels of dissolved oxygen. However, dilute solutions (e.g., 1 × 10^−3^ m at pH 7) will be completely oxidized to cystine in a day or so at room temperature, if exposed to the atmosphere. The 0.01 m solutions used in the studies reported here should not be affected by dissolved oxygen during the time scale of the experiments.

Column solution samples after 30 min of bubbling in octanoyl cysteine and also the solution remaining inside the column, after 90 min were sent for arsenic speciation determination analysis. As can be seen in **Table** **4**, after 30 min of ion flotation using the single‐chain octanoyl cysteine surfactant, about 99.47% of As(V) in the feed had been reduced to As(III) by the cysteine‐based surfactant and removed from the initial solution, which is consistent with results obtained by ICP‐MS. After 90 min, samples taken from inside the column showed that only a further 0.02% of As(V) (remaining in the column) was reduced to As(III), as the vast majority was reduced in the first 30 min.

**Table 4:** Speciation outcome for the ion flotation of arsenic solution (5320.6 µg L^−1^) in 0.01 m single‐chain octanoyl‐cysteine surfactant solution. All concentrations are µg L^−1.^

Sample	Total concentration	As(III)	As(V)	Total As(V) reduced to As(III)	Total As removal
In the waste collector after 30 min	5287.4	5259.4	27.9	99.47%	99.38%
Remaining in the flotation cell after 90 min	33.2	6.2	27.1	99.49%	99.38%

However, as there was no overflow of foam, in this case, after 30 min the As amount could not change beyond the removal level at 99.38%. This result illustrates that removing arsenic from the column solution occurred with a mutual oxidation/reduction reaction for cysteine to cystine and As(V) to As(III). Hence, the mechanism of binding could be either first by reduction and then binding or the other way around. Also, whether the binding was just with the sulfhydryl group or a chelating between the carboxyl and sulfhydryl group is not, at this stage, known. As(V) and As(III) ions are present to differing degrees in different parts of the World. For example, As(III) is dominant in Southeast Asia and As(V) in Argentina. Although we expect binding of cysteine to As(III), this was not studied in detail here.

In contrast, the double‐chain octanoyl cystine surfactant showed almost no (3%) arsenic removal after 30 min (see Table 3), possibly due to its low CMC and solubility, even though this surfactant did produce significant foaming and foam carryover for the first 30 min of the flotation process. This negative result may be a consequence of its low concentration (0.001 m) or because the arsenic ions only adsorb onto cysteine not cystine. After both 60 and 90 min, the arsenic remained in the column and no adsorption was observed. So, the octanoyl surfactants (both single‐ and double‐chain) showed good foaming but only the single‐chain was successful, producing 99% arsenic removal, whereas the double‐chain only gave 3% removal of arsenic.

The single‐chain octyl cysteine surfactant showed no arsenic adsorption (0%), for both 0.5 and 5 mg L^−1^ solution of arsenic, probably because it did not produce sufficient foam in the flotation system. It could also be because of the absence of an additional carbonyl group, which somehow affects the electronegativity of the normally adjacent NH group. If this is true, the hypothesis of a chelating mechanism in arsenic and cysteine binding is more likely.

The double‐chain dodecanoyl cystine surfactant showed no arsenic adsorption (0%) even though there was a significant level of foaming during the test and bubbles pushed out for the first 30 min of the experiment. It seems that this surfactant failed because of the small amount used (0.01 × 10^−3^ m), which was not sufficient for arsenic adsorption of 5 mg L^−1^. This negative outcome could also be because the arsenic adsorption by the cystine groups is prevented by spatial prohibition or because the doubled sulfur groups decrease the affinity of the cystine groups toward arsenic species in the solution. Moreover, lack of arsenic adsorption in this test is a proof of no artefactual processes in this test; that is, foaming alone is not sufficient without some specific surfactant head‐group adsorption mechanism.

### 2.4 Application of the Flotation Method in the Presence of Natural Lake Water, Using a Single‐Chain Octanoyl Cysteine Surfactant

The results of ICP‐MS analysis after 30, 60, and 90 min were 0.028 (relative standard deviation (RSD) of 4.14), 0.030 (11.81), and 0.024 (0.53), respectively; confirming successful arsenic adsorption with 99.43% adsorption after the first 30 min of running the flotation experiment using single‐chain octanoyl cysteine (C_11_H_21_NO_3_S). This result also demonstrated that this surfactant could retain its adsorbing features even in a complicated matrix, such as lake water, in which interfering ions such as Ca^2+^, Mg^2+^, and phosphate ions would also be present at similar or significantly higher levels than the As ions, at 5 ppm. After 30 min, there was no outflow; the adsorbed amount remained almost constant. In the blank chemical analysis of the lake water, 0.4 µg L^−1^ arsenic was found to be present, although this was ignored relative to the spiked 5000 µg L^−1^ arsenic level used in these experiments.

These lake water results were similar to the results obtained with purified water and purified single‐chain octanoyl cysteine (i.e., compare Tests 1 in Table 3), which suggests that removal efficiency is unaffected by using samples containing natural water.

It should also be noted that application of this process to natural water on a large scale, that is to produce large volumes of low arsenic drinking water, would involve the use of much taller columns than used in the laboratory study reported here. This would allow for a much high throughput rate of water flow, which would also reduce the running costs. We plan to develop a larger pilot scale unit to determine the real costs of this process.

Since the As‐cystine product in the collected foam, once destabilized, would have to be disposed of, for example, by encasement in cement and buried, the issue of the toxicity of the waste product is irrelevant. It will be important to demonstrate that only very low, insignificant levels of the complex exist in the product water. However, the octanoyl cysteine surfactant contains a peptide bond which is easily broken by natural enzymes (e.g., proteases) to produce octanoic acid (present in milk) and cysteine, which also occurs naturally.

## 3 Conclusions

The amino‐acid‐based surfactant octanoyl cysteine was synthesized and purified and found to successfully remove low levels of arsenic ions from water in a simple, single‐stage flotation system. In this system, the fluidity of the cysteine surfactant groups at the water–air interface should assist in effective chelation of the arsenic oxy‐ion species in the solution because it is expected that three to four cysteine groups are required for each As(III) or As(V) species. Once adsorbed, the arsenic can be effectively removed by the rising bubbles, which produced a maximum removal rate of 99.4–99.9% in a 5 mg L^−1^ feed solution, in a single‐stage process, and this indicates that the process is capable of reducing the arsenic content in natural level sources to a level lower than the recommended WHO limit of 0.01 mg L^−1^. Several other similar surfactants were synthesized and studied, as well as model compounds with partial cysteine properties. However, none of these gave the same level of removal as the single‐chain octanoyl cysteine. This compound and simple physicochemical process offers a potential new method for treating contaminated drinking water.

## 4 Experimental Section

*Materials: L‐*cysteine (97%), cystine, octanoyl chloride, dodecanoyl chloride, octyl isocyanate, octyl bromide, sodium octanoate (99%), octyl amine (99%), *tert‐*dodecylmercaptan (98.5%), myristyltrimethyl ammonium bromide (C_14_–TAB), thymolphthalein, arsenic standard solution (1000 mg L^−1^), sodium hydroxide, acetone, ethanol, methanol, and hexane were all purchased from Sigma‐Aldrich, Australia. All reagents were used without further purification. Milli‐Q water was used in all stages of these experiments.

**Figure 2:**
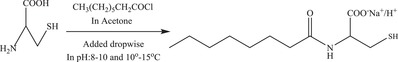
Scheme for the synthesis of single‐chain octanoyl cysteine.

*Synthesis of Single‐Chain Sodium Octanoyl Cysteine Surfactant (S‐octanoyl‐cys) and Double‐Chain Sodium Octanoyl Cystine Surfactant (D‐octanoyl‐cys)*: 0.06 mol of NaOH (0.04 mol for synthesis of *D‐*octanoyl‐cys) and 0.06 mol of *L‐*cysteine (0.02 mol *L‐*cystine for *D‐*octanoyl‐cys) were dissolved into 20 mL water (and into 100 mL of acetone and water 2:1 mixture for *D‐*octanoyl‐cys) at room temperature, followed by adding a mixture of 0.08 mol octanoyl chloride and 20 mL acetone (just 0.05 mol octanoyl chloride for *D‐*octanoyl‐cys), dropwise, while stirring at 10–15 °C. The pH of the solution was kept at about 8–10 by adding about 2 mL of sodium hydroxide solution (10%) before adding the mixture of octanoyl chloride and acetone and meanwhile (the mixture was stirred half an hour for the *D‐*octanoyl‐cys) 50 mL acetone was added to the resulting mixture of single chain octanoyl cysteine and then the precipitate was filtered and washed with acetone. The precipitate was then recrystallized two times (and three times for the *D‐*octanoyl‐cys) in a mixture of acetone:water (V:V, 50:50). This method followed the work of refs. 15 and^16^. A scheme for the synthesis of the single‐chain cysteine surfactants (e.g., C_8_–C_12_) is shown in **Figure** **2**.

The surfactants of interest would be formed from octanoyl, decanoyl, or dodecanoyl chloride. (Note that *S‐* refers to single‐chain and *D‐* refers to double‐chain).

*Synthesis of Single‐Chain Sodium Dodecanoyl Cysteine Surfactant (S‐dodecanoyl‐cys) and Double‐Chain Sodium Dodecanoyl Cystine Surfactant (D‐dodecanoyl‐cys)*: 0.02 mol of NaOH (0.04 mol for *D‐*dodecanoyl‐cys) and 0.02 mol of *L‐*cysteine (*L‐*cystine for *D‐*dodecanoyl‐cys) were dissolved into 100 mL of acetone and water (2:1) mixture at room temperature, followed by adding a mixture of 0.025 mol dodecanoyl (lauroyl) chloride (0.05 mol for *D‐*dodecanoyl‐cys), dropwise, while stirring at 10–15 °C. The pH of the solution was kept at about 8–10 by adding sodium hydroxide solution (10%). The mixture was stirred for half an hour, and then the precipitate was filtered and washed with acetone. The precipitate was then recrystallized two times (three times *D‐*dodecanoyl‐cys) in a mixture of ethanol:water (V:V, 95:5).^15^

**Figure 3:**
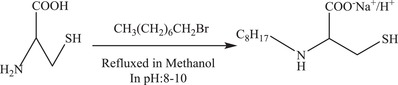
Scheme for the synthesis of single‐chain octyl cysteine.

*Synthesis of Single‐Chain Sodium Octyl Cysteine Surfactant (S‐octyl‐cys)*: 0.04 mol of NaOH and 0.04 mol of *L‐*cysteine were dissolved into 40 mL of methanol at 30 °C, followed by adding a small amount of thymolphthalein. 0.045 mol of octyl bromide was added to this mixture and refluxed for 5 h under alkaline conditions by adding NaOH (such that there was no change in blue color of the solution in the presence of thymolphthalein). The solution was left overnight. After solvent evaporation under reduced pressure, the residue was dissolved in water and its pH was decreased to about 5 by adding HCl 0.1 m. The precipitate formed was then filtered and washed with acetone, hexane and methanol and recrystallized two times in methanol.[[qv: 12a]] A scheme for the synthesis of the single‐chain octyl cysteine is shown in **Figure** **3**.

**Figure 4:**
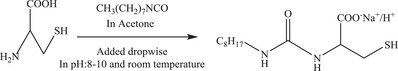
Scheme for the synthesis of single‐chain octyl isocyanate cysteine.

*Synthesis of Single‐Chain Sodium Octyl Isocyanate Cysteine Surfactant (S‐octyl isocyanate‐cys)*: 0.02 mol of NaOH and 0.02 mol of *L‐*cysteine were dissolved into 20 mL water at room temperature. After 1 h stirring, a mixture of 0.02 mol octyl isocyanate and 20 mL acetone was added to it, dropwise. The solution was then left overnight and the precipitate which was produced was washed with acetone and recrystallized two times in a mixture of acetone:water (V:V, 90:10).[[qv: 16c]] A scheme for the synthesis of the single‐chain octyl cysteine is shown in **Figure** **4**.

*Synthesis of Double‐Chain Sodium Octyl Isocyanate Cystine Surfactant (D‐octyl isocyanate‐cys)*: 0.02 mol of NaOH and 0.01 mol of *L‐*cystine were dissolved into 20 mL water at room temperature. After 1 h stirring, a mixture of 0.02 mol octyl isocyanate and 20 mL acetone was added to it, dropwise. The solution was then left overnight and the precipitate produced was filtered and washed with acetone and recrystallized two times in a mixture of acetone:water (V:V, 90:10).[[qv: 16b]]

*Mixture of Cysteine Functional Groups*: A mixture of 0.002 m of *tert*‐dodecyl mercaptan and C_14_–TAB (myristyltrimethyl ammonium bromide) was made using 0.001 m of each and Milli‐Q Water.

A mixture of the three functional groups also was made using sodium octanoate 0.001 m, octyl amine 0.001 m, and *tert‐*dodecylmercaptan 0.001 m, a total of 0.003 m.

*Product Characterization Methods*: The basic characterization of the synthesized surfactants was carried out using elemental analysis, CMC, and melting point determinations.

The octanoyl cysteine product was also characterized by ^1^H NMR spectroscopy and FT‐IR spectroscopic analysis. ^1^H spectroscopy was measured in D_2_O on an Oxford NMR 400 spectrometer operating at 400 MHz.

IR spectrum was obtained for samples contained in KBr pellets, using a Shimadzu IRPrestige‐21 Spectrophotometer.

Elemental analysis for the synthesized surfactants was carried out using an Elemental Analyzer, Model PE2400 CHNS/O (PerkinElmer, Shelton, CT, USA).

An ICP‐MS (Perkin Elmer, NexION 300D with Universal cell technology) was used to determine the arsenic solution concentrations.

Melting points for surfactant samples were measured using an Electrothermal IA9100 melting apparatus.

The As(V)/As(III) speciation measurements were carried out using a PE 200 Series high‐performance liquid chromatography (HPLC), using a PRPX 100 Column, attached to a polyethylene (PE) DRC‐e (Dynamic Reaction Cell–e) ICP‐MS. The plasma conditions used were: 1500W radio frequency (RF), at a gas flow rate of 0.88 mL min^−1^.

The conductivity and pH measurements were obtained using a Bench‐Top water quality meter SPER SCIENTIFIC Instrument model 860033.

*Ion Flotation Method—A Comparison of the Surfactants' Solution Properties*: The synthesized surfactants were compared to each other with respect to their water solubility, conductivity, critical micelle concentration, and foaming ability to determine basic suitability for the ion flotation process.

The conductivity for sodium octanoyl cysteine and cystine surfactant solutions were determined in alkaline water at pH = 9 at 25.0 °C, so that the surfactants were present as Na salts.

The conductivity for sodium dodecanoyl cysteine and cystine surfactants were also determined in alkaline water at pH = 7.5 for single‐chain and 9 for the double‐chain surfactant at 40.0 °C.

Foaming tests for each surfactant consisted of purging nitrogen gas through the flotation cell containing the synthesized surfactant solution (100 mL) in a range of 0.001–0.01 m, at gas flow rates of 1–5 L min^−1^ and checking the volume of the foam.

The solubility of each surfactant was initially examined by simply dissolving them in distilled water until an excess of solid was observed.

*Ion Flotation Method—Flotation System*: In a typical experiment, 0.01 m of the single‐chain octanoyl cysteine surfactant was dissolved (with stirring and heating to not more than 65 °C) in 50 mL of a slightly alkaline solution and then spiked with arsenic solution and made up to 100 mL to produce 5 mg L^−1^ solution of arsenic (as HAsO_4_^2−^) at a pH of about 8, using NaOH and Milli‐Q water (i.e., 10 mL of 50 mg L^−1^ of arsenic standard solution). Note that the 5 mg L^−1^ refers to the arsenic metal content in the solution.

The solution was then poured into a column of 30 cm height and 3 cm diameter, while a 3 L min^−1^ flow of nitrogen gas was passed through it (dried air was also used for the single‐chain octanoyl cysteine surfactant flotation for comparison). Two samples were taken from the bottom of the column, 50 mm above the sinter (glass sinter of size 2), after each of 30, 60, and 90 min, and the arsenic concentration of each sample were determined by ICP‐MS analysis. The upper‐outlet foam was also collected in a waste container using an outlet tube. A schematic diagram of the column setup is shown in Figure 1. The inlet is located halfway to prevent foam carry over. Due to the foaming characteristic of different surfactants the gas flow was varied.

0.001 m of the double‐chain octanoyl cystine surfactant and 0.01 × 10^−3^ m of double‐chain dodecanoyl cystine surfactant (equal to its CMC) were used separately in the same process with 5 mg L^−1^ arsenic solution and with nitrogen gas flow of 5 L min^−1^.

0.001 m single‐chain octyl cysteine surfactant was also studied in 0.5 and 5 mg L^−1^ arsenic solutions with a flow of 5 L min^−1^ of gas.

A mixture of sodium octanoate 0.001 m, *tert‐*dodecyl mercaptan 0.001 m, and octylamine 0.001 m in a total concentration of 0.003 m and another solution of 0.001 m of *tert‐*dodecyl mercaptan and 0.001 m of C_14_–TAB in a total concentration of 0.002 m were also used in the flotation column for evaluation of arsenic adsorption with the gas flow of 3 and 5 L min^−1^, respectively.

*Ion Flotation Method—Application of the Flotation Method in Natural Lake Water*: 0.01 m of the single‐chain octanoyl cysteine surfactant was dissolved (with stirring and heating to about 65 °C) in 50 mL of slightly alkaline lake water, which was spiked with 10 mL of 50 mg L^−1^ arsenic standard solution before starting the next step. 0.1 m of NaOH was added to keep the pH at about 8, and the solution made up to 100 mL of 5 mg L^−1^ arsenic solution (as HAsO_4_^2−^) using lake water.

The solution was then poured into the same column, while a 3 L min^−1^ flow of nitrogen gas was passed through it. Two samples were taken after each 30, 60, and 90 min and the arsenic concentration of each sample determined by ICP‐MS. The upper‐outlet foam was also collected in a waste container using an outlet tube.

## Conflict of Interest

The authors declare no conflict of interest.

## Supporting information

Supplementary
