# Supplementary material for: Low‐Level Arsenic Removal from Drinking Water
Source: Glob Chall. 2018 Nov 4;3(3):1700047. doi: 10.1002/gch2.201700047 (PMC6436586; doi:10.1002/gch2.201700047)
Supplement: Supplementary file 1 — Supplementary [file GCH2-3-1700047-s001.pdf]

Copyright WILEY-VCH Verlag GmbH & Co. KGaA, 69469 Weinheim, Germany, 2018.

Supporting Information

for *Global Challenges,* DOI: 10.1002/gch2.201700047

Low-Level Arsenic Removal from Drinking Water

Fatemeh Makavipour, Richard M. Pashley, and A. F. M. Mokhlesur Rahman*

Copyright WILEY-VCH Verlag GmbH & Co. KGaA, 69469 Weinheim, Germany, 2016.

Supporting Information

Low-Level Arsenic Removal from Drinking Water

Fatemeh Makavipour, Richard M. Pashley and A.F.M. Mokhlesur Rahman
School of Physical, Environmental & Mathematical Sciences, UNSW Canberra, Australia.
***Corresponding author: Professor Ric Pashley, r.pashley@adfa.edu.au; School of Physical, Environmental & Mathematical Sciences, UNSW Canberra, Northcott Drive, Canberra, ACT 2610, Australia.**

Supporting Information

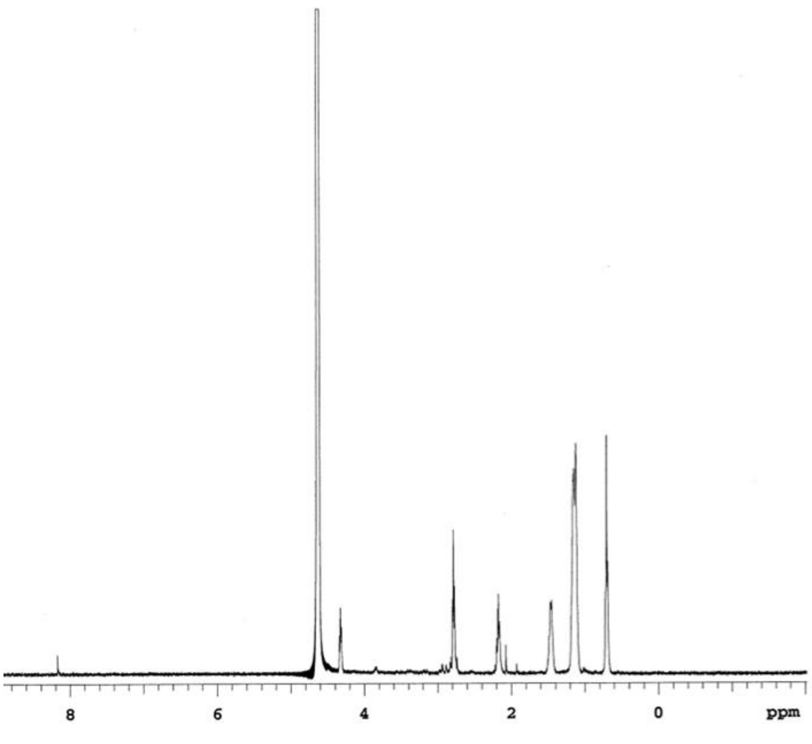

Figure S1. ^{1}HNMR spectrum of twice-recrystallized octanoyl cysteine surfactant.

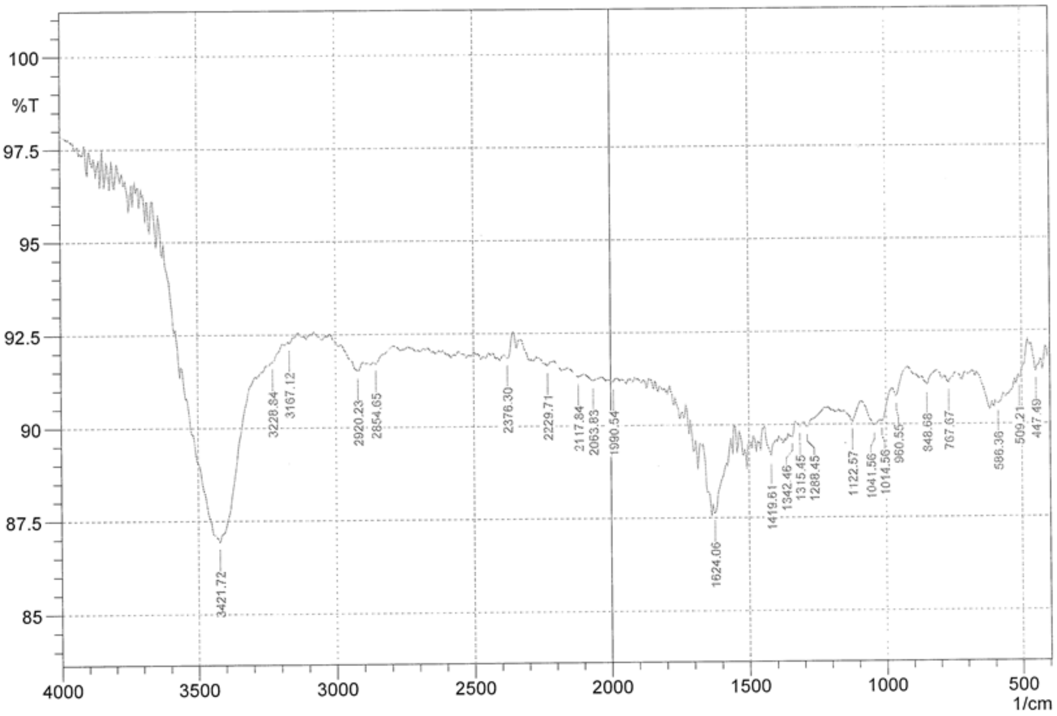

Figure S2. FT-IR spectrum of double-recrystallized octanoyl cysteine surfactant.

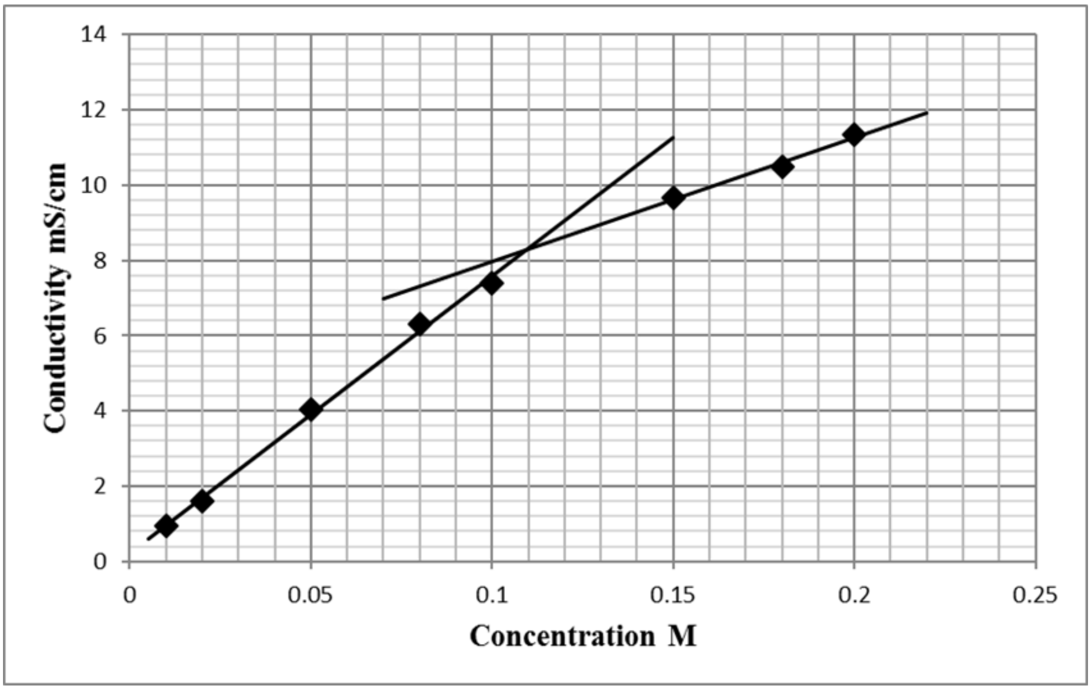

Figure S3. Variation of conductivity with single-chain surfactant concentration (*S*-octanoyl-cys) in alkaline solution at pH=9, 25.0°C.

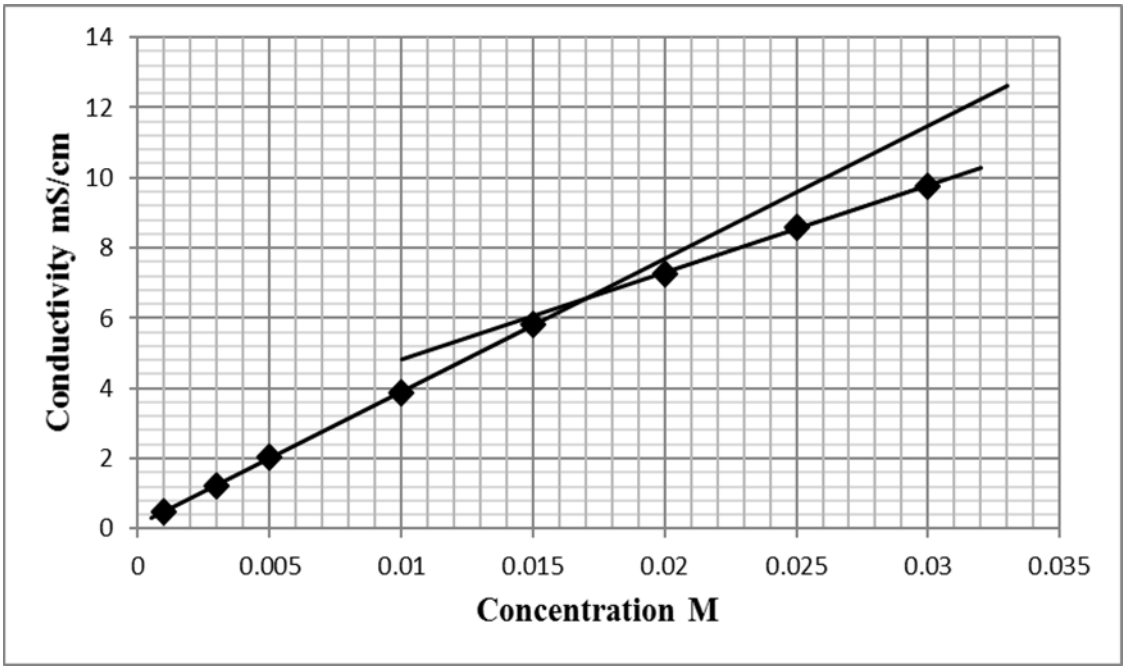

Figure S4. Variation of conductivity with double-chain surfactant concentration (*D*-octanoyl-cys) in alkaline solution at pH=9.5, 25.0°C.

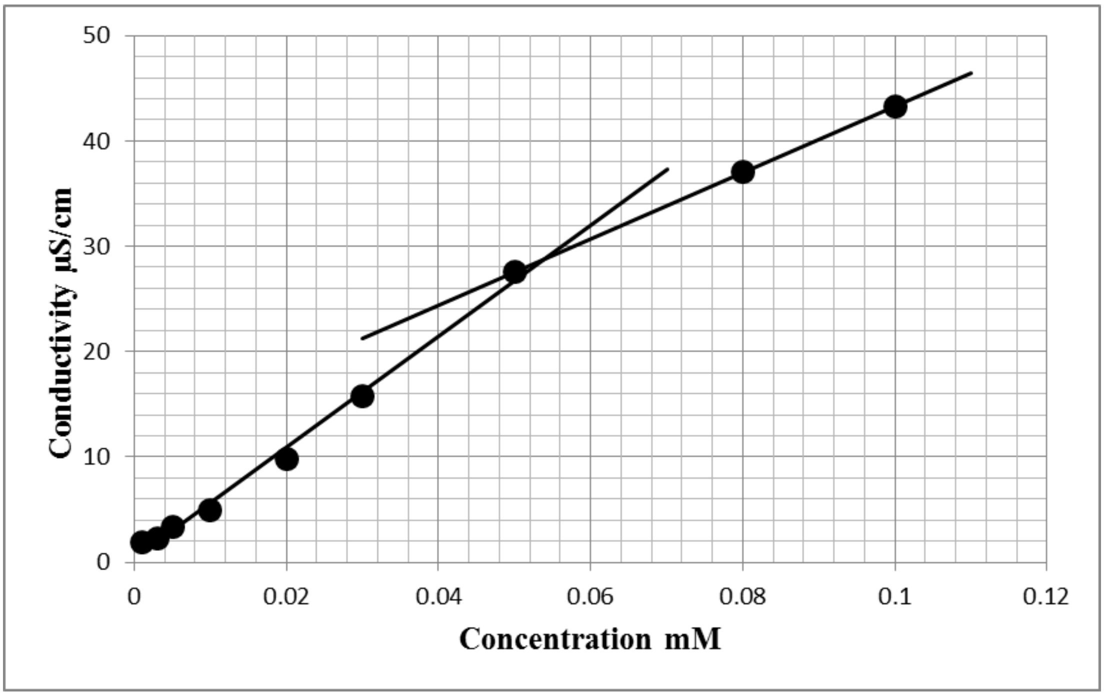

Figure S5. Variation of conductivity with single-chain surfactant concentration (*S*-dodecanoyl-cys) in alkaline solution at pH=7.5, 40.0°C.

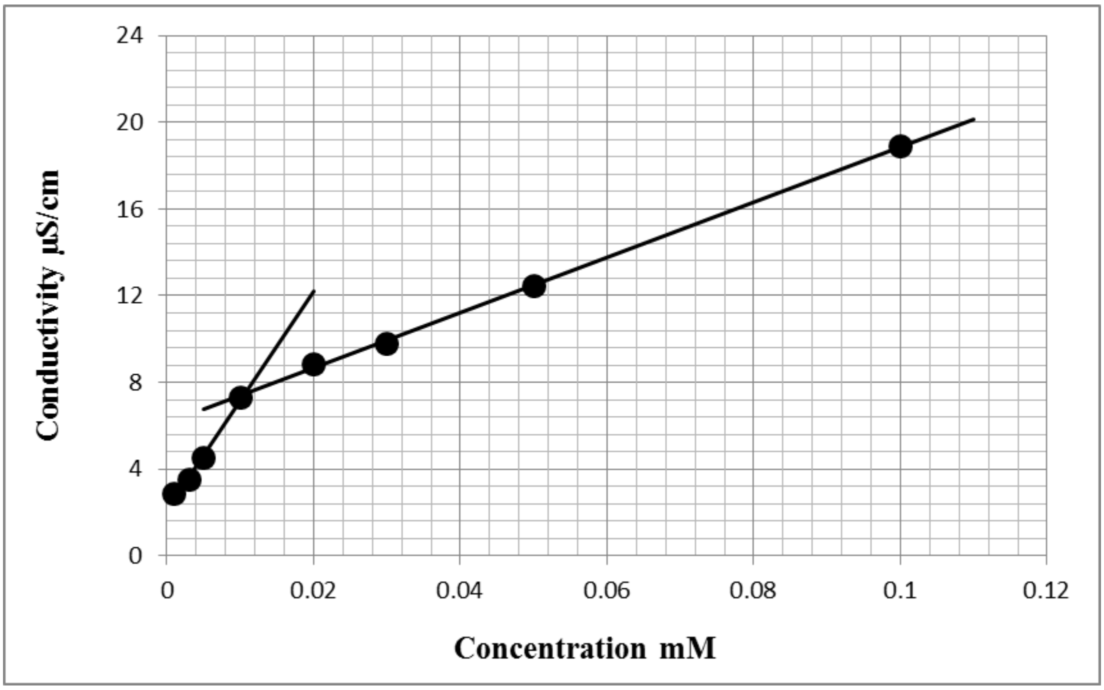

Figure S6. Variation of conductivity with double-chain surfactant concentration (*D*-dodecanoyl-cys) in alkaline solution at pH=9, 40.0°C.
